# Quantitative analysis of insulin-like growth factor 2 receptor and insulin-like growth factor binding proteins to identify control mechanisms for insulin-like growth factor 1 receptor phosphorylation

**DOI:** 10.1186/s12918-016-0263-6

**Published:** 2016-02-09

**Authors:** Dan Tian, Isaiah Mitchell, Pamela K. Kreeger

**Affiliations:** Department of Biomedical Engineering, 4553 WI Institute Medical Research, University of Wisconsin-Madison, 1111 Highland Ave, Madison, WI 53705 USA; University of Wisconsin Carbone Cancer Center, 600 Highland Ave, Madison, WI 53792 USA

**Keywords:** Insulin-like growth factor (IGF), Mathematical modeling, Ovarian cancer

## Abstract

**Background:**

The insulin-like growth factor (IGF) system impacts cellular development by regulating proliferation, differentiation, and apoptosis, and is an attractive therapeutic target in cancer. The IGF system is complex, with two ligands (IGF1, IGF2), two receptors (IGF1R, IGF2R), and at least six high affinity IGF-binding proteins (IGFBPs) that regulate IGF ligand bioavailability. While the individual components of the IGF system are well studied, the question of how these different components integrate as a system to regulate cell behavior is less clear.

**Results:**

To analyze the relative importance of different mechanisms that control IGF network activity, we developed a mass-action kinetic model incorporating cell surface binding, phosphorylation, and intracellular trafficking events. The model was calibrated and validated using experimental data collected from OVCAR5, an immortalized ovarian cancer cell line. We then performed model analysis to examine the ability of IGF2R or IGFBPs to counteract phosphorylation of IGF1R, a critical step for IGF network activation. This analysis suggested that IGF2R levels would need to be 320-fold greater than IGF1R in order to decrease pIGF1R by 25 %, while IGFBP levels would need to be 390-fold greater. Analysis of The Cancer Genome Atlas (TCGA) data set suggested that this level of overexpression is unlikely for IGF2R in ovarian, breast, and colon cancer. In contrast, IGFBPs can likely reach these levels, suggesting that IGFBPs are the more critical regulator of IGF1R network activity. Levels of phosphorylated IGF1R were insensitive to changes in parameters regulating the IGF2R arm of the network.

**Conclusions:**

Using a mass-action kinetic model, we determined that IGF2R plays a minor role in regulating the activity of IGF1R under a variety of conditions and that due to their high expression levels, IGFBPs are the dominant mechanism to regulating IGF network activation.

**Electronic supplementary material:**

The online version of this article (doi:10.1186/s12918-016-0263-6) contains supplementary material, which is available to authorized users.

## Background

The insulin-like growth factor (IGF) network plays important roles in homeostasis and disease by mediating cell proliferation, differentiation, and apoptosis [1]. Elevated levels of IGF ligands have been observed in ovarian, breast, and colon cancer, and are associated with poor prognosis and resistance to treatment [2–6]. Therefore, the IGF network has been proposed as a potential therapeutic target that could benefit a broad group of patients. However, clinical trials with IGF-targeted inhibitors have had mixed results [7]. While the individual elements of the network have been well characterized, it is less clear how these components interact, information that may provide new methods to better counteract IGF-mediated effects in cancer.

The IGF network is composed of two ligands (IGF1, IGF2), two receptors (IGF1R, IGF2R), and a family of six high affinity binding proteins (IGFBPs) [8]. IGF1 and IGF2 are produced in the liver and other tissues, and act both locally and following circulation through the bloodstream to reach target tissues. *Igf1−/−* and *Igf2−/−* mice were significantly smaller than their wildtype littermates, displayed severe muscle dystrophy and died perinatally [9, 10]. IGF1R is a receptor tyrosine kinase that binds IGF1 and IGF2 to initiate a cascade of downstream signaling pathways such as the MAPK/ERK and PI3K/AKT. Similar to other receptor tyrosine kinases, IGF-IGF1R complexes are internalized by receptor-mediated endocytosis and degraded by the lysosome or recycled back to the cell surface [11]. *Igf1r−/−* mice exhibited severe growth restriction and died shortly after birth of respiratory failure [12]. Additionally, IGF1R and the closely related insulin receptor (IR) form signaling-competent heterodimers of IGF1R/IR hybrid receptors in cells that express both receptors [13]. IGF2R specifically binds IGF2, but lacks an intracellular tyrosine kinase domain [14]. While IGF2R cannot initiate downstream signaling cascades, IGF2-IGF2R complexes undergo cellular trafficking, potentially regulating extracellular IGF2 levels and providing an indirect mechanism to influence cellular behavior [11]. Consistent with this, *Igf2r−/−* mice exhibited increased levels of IGF2 and died perinatally due to abnormal growth [15, 16]. In addition to these interactions, circulating levels of IGF1 and IGF2 are regulated by high-affinity interactions with IGFBPs [17]. These interactions increase ligand stability and utilize some of the same residues as the ligand-receptor interaction, leading to competitive inhibition [18].

The balance of these different IGF network components (i.e.*,* ligands, binding proteins, receptors) likely plays an important role in maintaining healthy tissue. For example, elevated *IGF1* and *IGF2* expression were linked to disease progression and poor survival in ovarian cancer [2, 3]. Additionally, differences in receptor and binding protein levels have been reported [19, 20], but in contrast to other receptor systems, dramatic overexpression or mutations that impact protein function appear to be rare [7]. Therefore, it will be important to better understand how the more subtle balances between these different components influence network activity. In particular, IGFBPs and IGF2R provide two separate mechanisms to regulate IGF2 bioavailability and have each been suggested as a potential tumor suppressor [21–23]; however, it remains unclear which plays the dominant role in regulating IGF2 activity in tumors. Computational modeling is a useful method to analyze how changes in individual components impact network activity, and has been valuable in understanding the impact of other signaling networks on tumor development, progression, and treatment [24]. Most of the prior models of the IGF network have focused exclusively on IGF1R and have not incorporated the impact of IGF2R or IGFBPs [25, 26]. We have previously developed a computational model of the interactions between IGF1, IGF1R, and IGFBPs in ovarian cancer cells [27]. Analysis of this model suggested, and experimental results confirmed, that IGFBPs were key regulators of IGF1-mediated IGF1R activation. In addition, a more complete model of the IGF system in cartilage has been developed, incorporating extracellular and cell surface interactions of IGF1, IGF2, IGFBPs, IGF1R, and IGF2R [28]. Model analysis suggested that IGF2R levels could influence IGF1R activation; however, the impact of changes in the level of IGFBPs was not examined and this analysis was not experimentally validated. Therefore, to analyze the relative role of IGFBPs and IGF2R in cancer, we developed a mass-action kinetics model of the IGF network that incorporated IGF1, IGF2, IGF1R, IGF2R, and IGFBPs. The model was calibrated and validated using an ovarian cancer cell line, OVCAR5, and then analyzed to determine the relative impact of IGF2R and IGFBPs on IGF2-mediated IGF1R activation.

## Results and discussion

### A calibrated mass-action kinetic model accurately captures pIGF1R dynamics

**Fig. 1:**
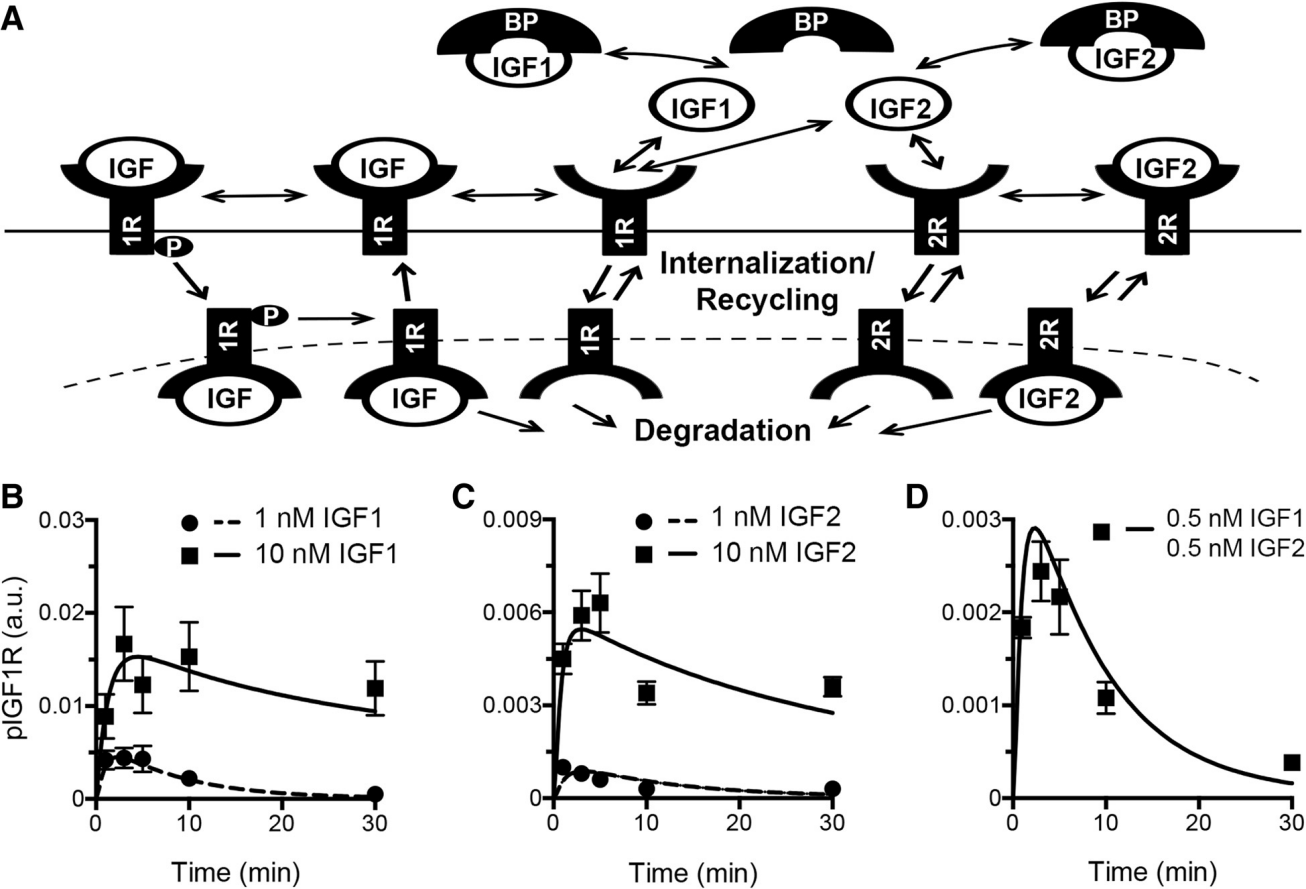
The IGF network model qualitatively and quantitatively captured pIGF1R experimental dynamics. **a** Diagram of molecular events included in the model, double-sided arrows indicate reversible events. **b**, c Comparison of the best-fit model to experimental results for IGF1- (**b**) and IGF2- (**c**) treated OVCAR5. **d** The calibrated model was used to predict the effect of a mixture of 0.5 nM IGF1 and 0.5 nM IGF2 on pIGF1R in OVCAR5. In these experiments, IGF2 was added directly to the culture media, which contained OVCAR5-secreted IGFBPs. Model results are represented by solid lines and experimental data are represented by circles or squares indicating average ± SD, *n* = 3

To examine which factors regulate IGF1R phosphorylation, and therefore IGF network activation, we developed a mass-action kinetic model that incorporated cell surface interactions and intracellular trafficking events following treatment with IGF1 and/or IGF2 (Fig. 1a). Details about the model structure and parameters can be found in Additional file 1. Where possible, parameter values were obtained from literature or experimental analysis of OVCAR5 cells (Additional file 2); remaining parameters were fit to experimental timecourses of phosphorylated IGF1R (pIGF1R) in OVCAR5 cells stimulated with 1 nM IGF1, 10 nM IGF1, 1 nM IGF2, or 10 nM IGF2 (Fig. 1b, c).

The experimental data demonstrated that after ligand treatment, there was a rapid and transient peak in pIGF1R (Fig. 1b, c). For treatment with high doses (10 nM IGF1 or IGF2), the level of pIGF1R remained above 50 % of the peak level at 30 min; in contrast, treatments with lower doses (1 nM IGF1 or IGF2) did not result in sustained activation. Additionally, IGF1 treatment was observed to elicit a stronger peak in pIGF1R than IGF2 treatment for the same dose, consistent with previous reports [29]. The experimental data was well-described by the model solution, with the timing and magnitude of the pIGF1R peak accurately captured, suggesting that the model framework adequately described the dynamics of the IGF system in response to either stimuli. We next examined the model’s ability to predict the impact of simultaneous exposure to IGF1 and IGF2, as cells in vivo are frequently exposed to both stimuli. OVCAR5 were treated with a mixture of 0.5 nM IGF1 and 0.5 nM IGF2 and pIGF1R was measured as a function of time. Experimental results demonstrated that this mixture led to signaling that was intermediate in magnitude relative to 1 nM of the individual ligands, suggesting that the system behaves in a linear fashion when below saturating levels. Comparison of model predictions and experimental data demonstrated good agreement (Fig. 1d), validating the model’s predictive capability.

### Changes in IGF2R levels do not affect pIGF1R magnitude or dynamics

**Fig. 2:**
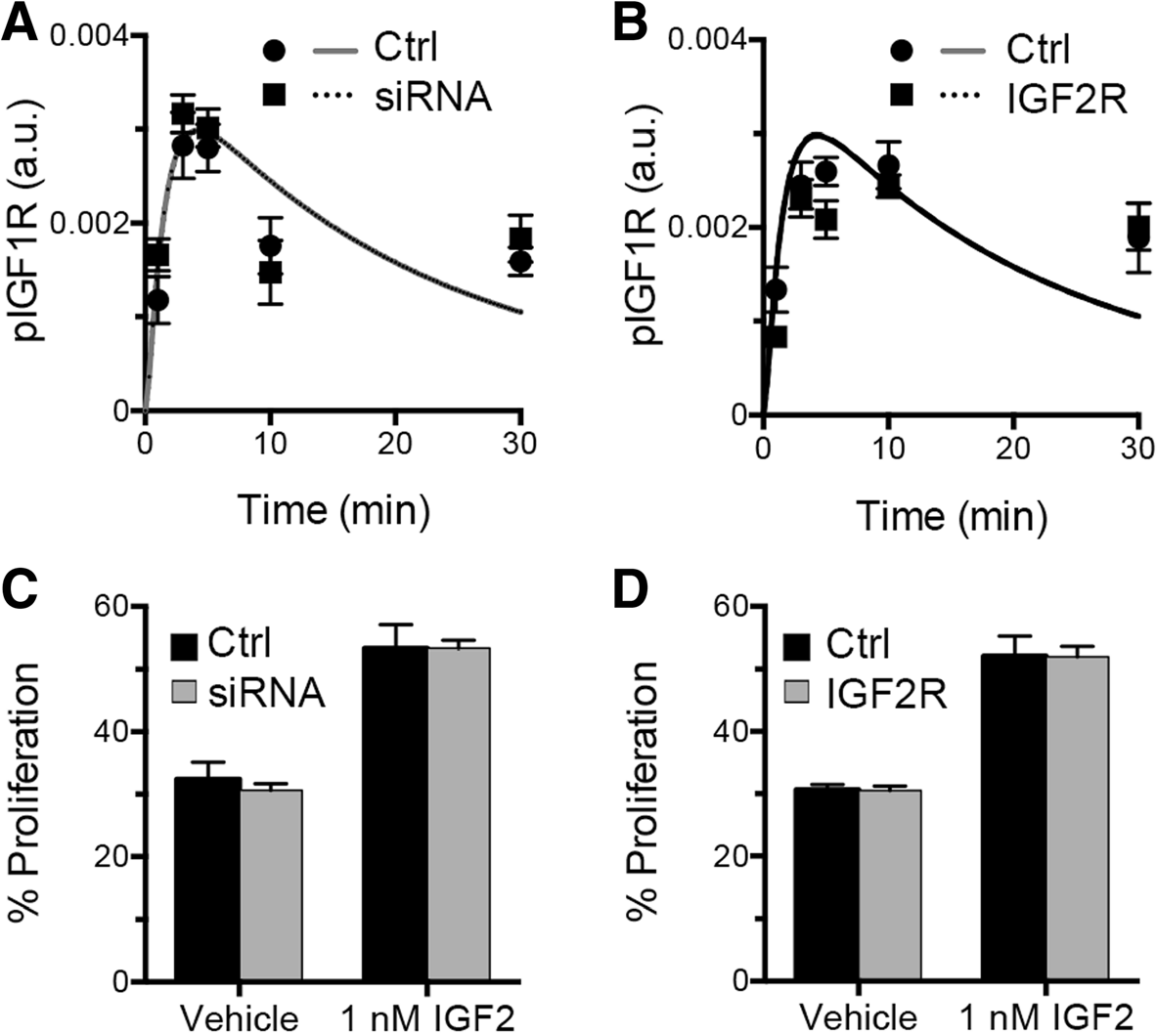
Alterations in IGF2R levels did not substantially impact phosphorylation of IGF1R or cell proliferation. **a**, **b** Model results predicted that a complete knockdown (**a**) or a 1.4-fold overexpression (**b**) of IGF2R in OVCAR5 would not affect pIGF1R. This prediction was consistent with experimental data collected from these conditions. In these experiments, IGF2 was added to fresh serum-free media, and therefore did not contain OVCAR5-secreted IGFBPs. **c**, **d** IGF2-induced proliferation was not impacted by knockdown of IGF2R (**c**) nor a 1.4-fold overexpression of IGF2R (**d**), *p* > 0.05

IGF2R has been postulated to act as a tumor suppressor by binding and clearing IGF2, thereby preventing activation of IGF1R [22]. To better understand the influence of IGF2R on the IGF network, we utilized the model to examine the effect of changes in IGF2R levels on IGF2-induced phosphorylation of IGF1R. Simulations were performed for conditions corresponding to 1 nM IGF2 and the level of IGF2R knockdown and overexpression that was achieved experimentally in OVCAR5 (Fig. 2, Additional file 3). To isolate the effects of IGF2R, simulations were conducted assuming no IGFBPs were present and experiments were performed in fresh serum-free media to minimize cell-secreted IGFBPs [27]. While IGF2R sequestration of IGF2 is expected to regulate IGF1R activation [30], the validated model predicted that IGF2R knockdown would have no discernable effect. The experimental results confirmed this prediction, with no significant difference in pIGF1R at all time points for either knockdown (Fig. 2a) or overexpression (Fig. 2b). As our model only captured early time dynamics, we tested the impact of knockdown and overexpression of IGF2R on IGF2-induced proliferation and determined that this change to the IGF network had no significant long-term effects as well (Fig. 2c, d). In contrast, prior studies in breast cancer cells showed that decreased IGF2R expression resulted in a higher growth rate [31] while increased IGF2R expression led to decreased invasiveness and motility [32]. However, these prior studies did not measure either the absolute level of IGF receptors or the ratio of IGF2R to IGF1R, which may significantly affect IGF network activation.

**Fig. 3:**
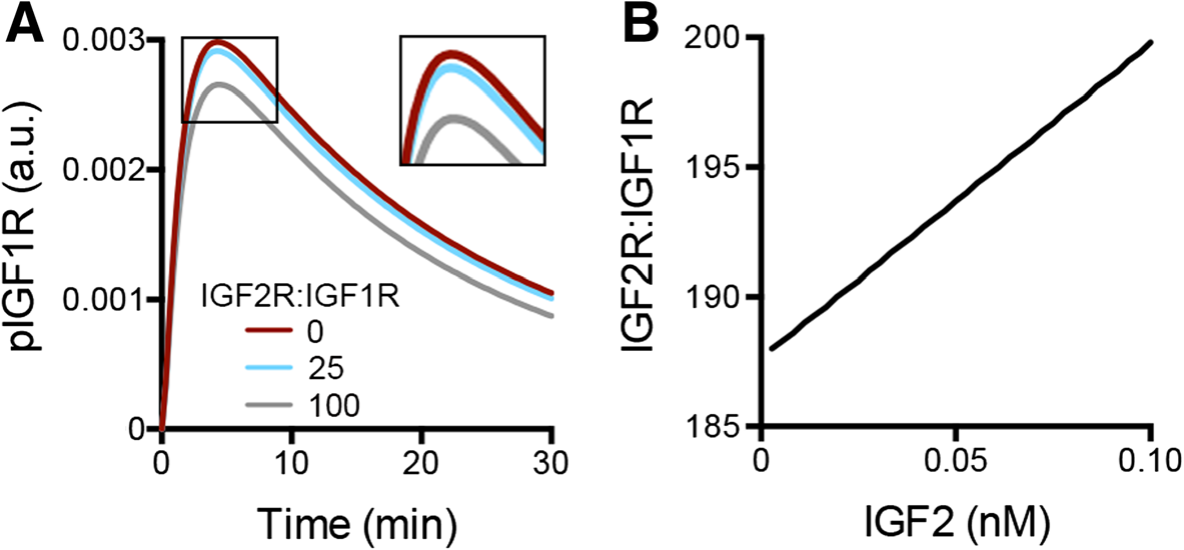
Model results suggest that IGF2R can suppress phosphorylation of IGF1R for specific quantitative combinations. **a** Model analysis demonstrated that IGF2R would need to be 100 times more abundant than IGF1R in order to inhibit pIGF1R by 11.2 % in OVCAR5. **b** Increasing IGF2 treatment doses increased the magnitude of IGF2R:IGF1R ratio required for 50 % inhibition of pIGF1R

Therefore, we next utilized the model to determine what ratio of IGF2R:IGF1R was needed for IGF2R to be an effective suppressor of IGF2-mediated activation. The model was utilized to simulate the effects of overexpressing IGF2R relative to IGF1R, with IGF2R:IGF1R ranging from 1:1 to 100:1. As shown in Fig. 3a, an IGF2R:IGF1R ratio of 100 would be necessary to reduce the peak activation of IGF1R by 11.2 %. In the experimental validation, IGF2R was overexpressed by 1.4-fold, resulting in an IGF2R:IGF1R ratio of approximately 1:1. As the IGF2R:IGF1R ratio needed for substantial reduction in pIGF1R was dramatically higher than what was achieved in OVCAR5, it was unsurprising that the IGF2R overexpression had minimal impact experimentally (Fig. 2). We next interrogated the TCGA database for measurements of IGF receptor expression to determine if this level of IGF2R overexpression is possible in tumor samples. A complete compendium of protein expression of the different IGF system components is not available through The Cancer Proteome Atlas (TCPA, [33]); in particular, the reverse phase protein array used for most samples in this database included IGFBP2 but did not measure IGF1R or IGF2R. Therefore, we utilized gene expression levels from RNA sequencing (RNA-Seq). While transcription levels are not expected to correlate precisely with protein levels, RNA-Seq data has been shown to be qualitatively consistent with proteomic measurements [34]. The TCGA database was utilized to compare the relative expression of *IGF2R* to *IGF1R* in three different carcinomas in which IGF activity has been reported to play a role (ovarian serous cystadenocarcinoma [35], breast invasive carcinoma [36], and colon adenocarcinoma [37]; Additional file 4). For all three tumor types, *IGF2R:IGF1R* was generally less than 10, with only a few tumors expressing as much as 25 times as much *IGF2R*. Thus, while it is possible for the IGF2R:IGF1R ratio to reach levels where the model would predict a substantial suppression of pIGF1R, it is expected that this would be rare in tumors. However, as a putative tumor suppressor, it is possible that IGF2R levels are decreased during tumor development, such that the ratio of the two receptors is altered in the pathophysiological state. The level of *IGF2R* was significantly lower in both breast and colon cancers compared to normal tissue (*p* < 0.01, data for normal tissue samples is not currently available in the TCGA database for ovarian cancer), consistent with prior reports of reduced *IGF2R* expression in hepatocellular carcinomas compared to normal liver tissue [38]. However, *IGF1R* expression was also significantly lower for breast tumors (*p* < 0.01). Therefore, since both receptor levels may change with tumor development, we also compared the *IGF2R*:*IGF1R* ratio between tumor and normal tissue (Additional file 4). Despite the decrease in *IGF2R* levels, this ratio was unchanged for colon cancers and was actually significantly higher for breast tumors relative to normal tissue, even though the ratio did not reach levels that our model predicts would impact signaling. Combined, the model analysis, experimental results, and TCGA data suggest that IGF2R does not play a substantial role in suppressing IGF2 in the examined tumor types. This does not preclude a role for IGF2R in other tumor types; for example, hepatocellular carcinomas have been found to have mutations in *IGF2R* [39].

Our previous study indicated that the extracellular level of IGF1 relative to the available IGF1R level was an important metric that impacted the activation of this network [27]. Therefore, we hypothesized that a potential factor accounting for the inefficacy of IGF2R suppression of IGF2 was the high amount of IGF2 relative to IGF receptors in OVCAR5. The IGF2 treatment dose of 1 nM was on the order of 10^−9^ nmol/cell while the experimentally determined concentration of IGF2R was on the order of 10^−11^ nmol/cell; therefore, even if every IGF2R sequestered an IGF2 ligand there would still be a substantial amount of IGF2 available to activate IGF1R. To examine if the effectiveness of IGF2R in suppressing pIGF1R was dependent on IGF2 dose, model analysis was performed for a range of IGF2 doses to determine the IGF2R:IGF1R ratio necessary to cut the peak pIGF1R level by 50 % (Fig. 3b). This model analysis suggests that for very low doses of IGF2, the amount of IGF2R that is needed to impact the system does decrease; however, even at doses as low as 0.01 nM IGF2, IGF2R would need to be 188.8 times as abundant as IGF1R in order to decrease pIGF1R activation to that extent. As physiological concentrations of free IGF2 range from 0.2 to 2 nM [40, 41], this analysis provides further support that IGF2R does not play a significant role in suppressing IGF network activity in most tumors.

### IGFBP levels impact levels of phosphorylated IGF1R

**Fig. 4:**
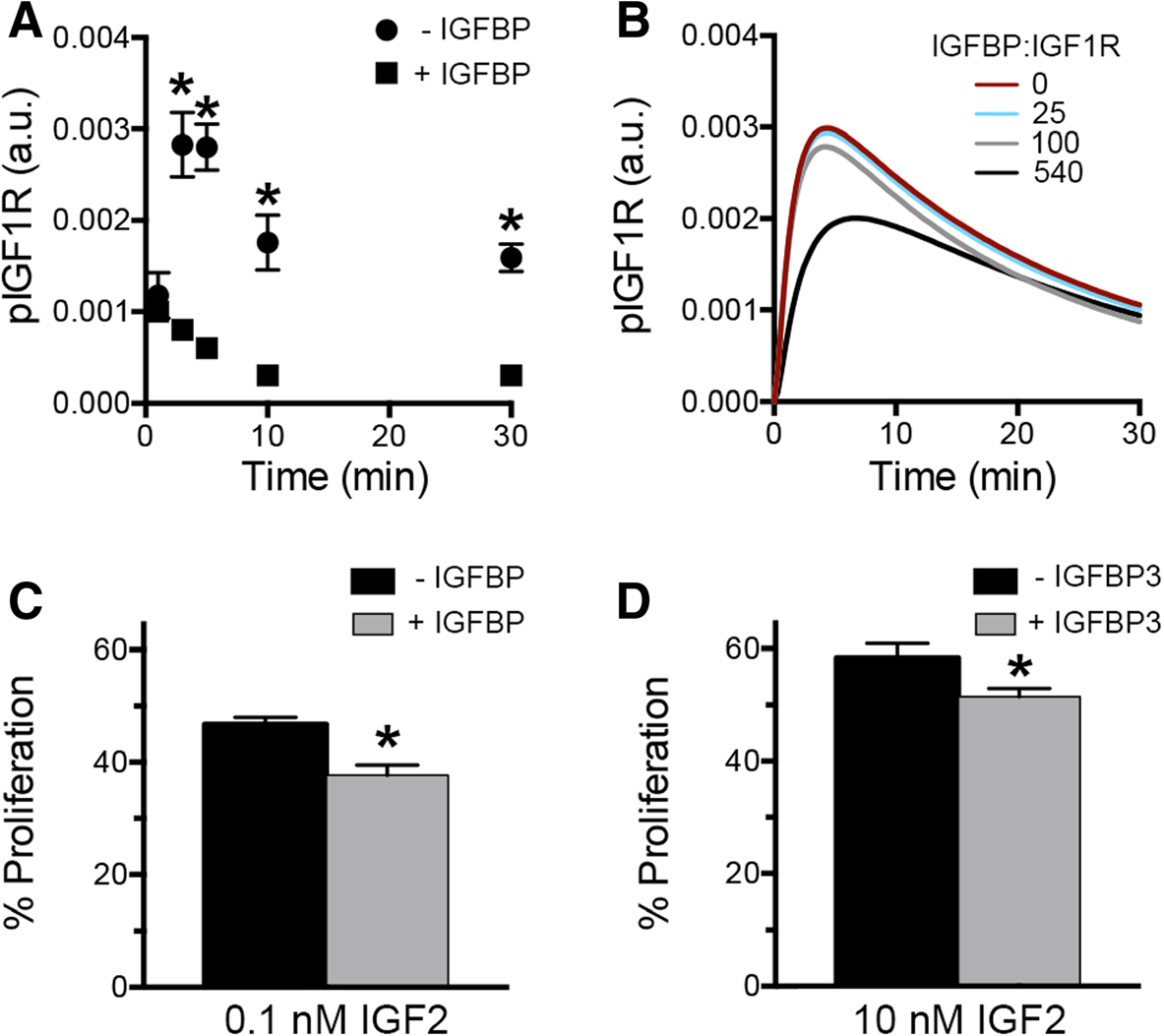
Model results suggest that IGFBP can suppress phosphorylation of IGF1R for specific quantitative combinations. **a** Treatment with IGF2 in the presence of OVCAR5-secreted IGFBPs resulted in significantly lower levels of pIGF1R, data replotted from Figs. 1c and 2a. **b** Model results demonstrated that IGFBP would suppress pIGF1R by 7 % when available at 100-fold the level of IGF1R. OVCAR5 levels of IGFBPs were 540-fold the level of IGF1R, and were predicted to substantially decrease pIGF1R. **c** IGF2-induced proliferation was significantly decreased in the presence of IGFBPs secreted by OVCAR5 cells. **d** IGF2-induced proliferation of OVCAR5 cells was significantly decreased in the presence of recombinant IGFBP3, * indicates *p* < 0.05

Our experimental results suggested that while IGF2R minimally impacted IGF1R network activity, the presence of IGFBPs substantially impacted pIGF1R levels, with peak activation increased by 3-fold when cell-secreted IGFBPs were removed from the media (Fig. 1c vs. Fig. 2a, replotted in Fig. 4a). Therefore, we next examined the role of IGFBPs in regulating IGF2-induced network activity. IGFBPs bind to circulating IGF1 or IGF2, sequestering the ligand from proteases and controlling its presentation to IGF1R [42]. While the regulatory role of IGFBPs in the interaction between IGF ligands and their receptors is well-known, there has been limited quantitative analysis of the effect of this function. When simulations were run for the baseline levels of IGF1R and IGF2R in OVCAR5, pIGF1R was found to decrease with increasing levels of IGFBP (Fig. 4b). Interestingly, IGFBPs were less effective in controlling phosphorylation of IGF1R than IGF2R when examined at the same ratio (Fig. 3a), since IGF2R can target the ligand for degradation while IGFBPs only sequester it. As a result, IGFBP expression would still need to be in large excess relative to IGF1R in order to play a substantial role in IGF network activity. The initial conditions for OVCAR5 indicate that for some tumor cell lines this is possible, as the ratio of IGFBP to IGF1R per cell was experimentally determined to be 543 (Additional file 1). Therefore, we examined the TCGA data to compare expression of *IGFBP* relative to *IGF1R* in ovarian, breast, and colon carcinoma (Additional file 5). In contrast to the moderate levels of *IGF2R:IGF1R*, the *IGFBP*:*IGF1R* ratios for all three carcinomas were frequently between 100 and 200, indicating that IGFBPs might play a more significant role than IGF2R in regulating the network response to IGF2. Our prior results demonstrated that IGFBP impacted IGF1-induced proliferation in OVCAR5 [27]; therefore, we examined if IGFBPs were sufficient to counteract IGF2 in vitro. OVCAR5 were treated with IGF2 in fresh serum-free media or in conditioned media containing IGFBPs. Proliferation was significantly decreased in the presence of IGFBPs, further suggesting that IGFBPs play a central role in regulating IGF network response to IGF2 (Fig. 4c). To further confirm that this inhibition resulted from IGFBPs and not another secreted factor in the conditioned media, OVCAR5 were pretreated with recombinant IGFPB3 prior to addition of IGF2, and a similar effect was observed (Fig. 4d). This effect resulted from both the high affinity of IGFBPs for IGF2 (Additional file 1) and the production and secretion of large quantities of IGFBPs relative to receptor levels. Importantly, IGFBP levels have been shown to vary between healthy and diseased states, providing a mechanism for IGF network activity to change during disease development. For example, IGFBP3 levels were inversely correlated with ovarian cancer risk [43] and breast cancer patients had reduced IGFBP-1, −3, and −6 serum levels compared to patients with benign tumors [44].

### Multivariate analysis further supports a limited role for IGF2R

**Fig. 5:**
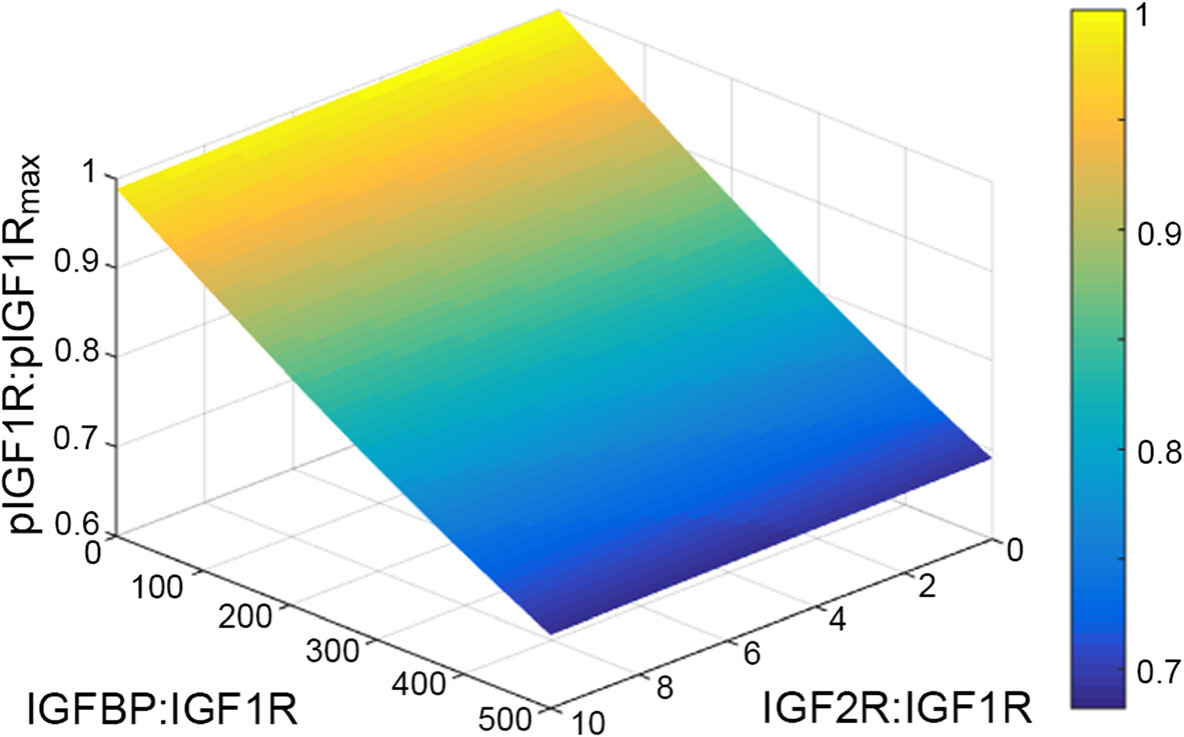
IGFBP and IGF2R demonstrated an additive effect when examined in combination. Model simulations of different combinations of IGF2R and IGFBP levels indicated that increases in IGFBP and IGF2R would act together, but would not synergize, to counteract IGF2-induced phosphorylation of IGF1R

As the TCGA data clearly demonstrated that tumors simultaneously express *IGF1R*, *IGF2R*, and *IGFBP*, we next examined the combined effect of IGF2R and IGFBP on phosphorylation of IGF1R in response to IGF2. Model simulations were run using combinations of IGF2R:IGF1R and IGFBP:IGF1R that spanned the range identified in the TCGA data set (0–10 for IGF2R, 0–500 for IGFBP). As seen in Fig. 5, these two elements combine in an additive fashion to regulate IGF1R activation. Therefore, while IGF2R was able to supplement the ability of IGFBP to prevent IGF1R activation, the dominant control mechanism at these levels is expected to remain extracellular ligand sequestration by IGFBPs.

**Fig. 6:**
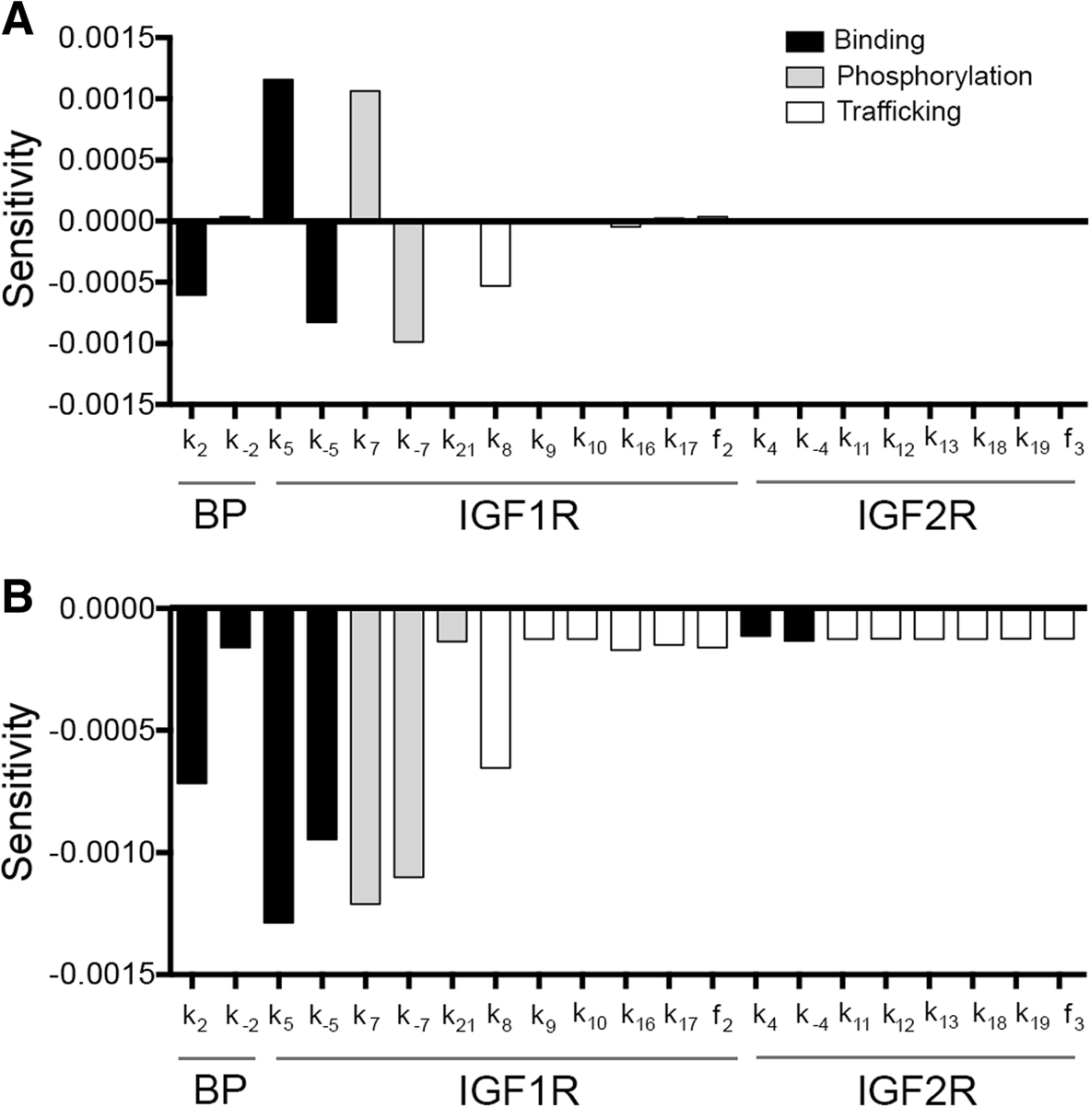
Sensitivity analysis demonstrated that pIGF1R was insensitive to changes in the IGF2R network. **a** Sensitivity of the integrated level of pIGF1R when individual rate parameters were changed. A positive value indicates an increase in the level of pIGF1R. **b** Sensitivity of the integrated level of pIGF1R when rate parameters are changed in combination with a 10-fold increase in the level of IGF2R. Parameters were altered such that both perturbations resulted in a decrease in the level of pIGF1R

It is possible that in other ovarian cancer cell lines or tumors, a change in one of the rate parameters could alter the network’s sensitivity to changes in the level of IGF2R. To examine this possibility, a sensitivity analysis was performed, examining first the effect of perturbations to the individual parameters (Fig. 6a) and then the effect of changes in the parameters in combination with a 10-fold increase in IGF2R (Fig. 6b). The single-parameter analysis indicated that the level of pIGF1R was most sensitive to parameters that impacted the rate of IGF1R phosphorylation/dephosphorylation (*k*_*7*_/*k*_*−7*_) or IGF2-IGF1R association/dissociation (*k*_*5*_/*k*_*−5*_). This sensitivity is not surprising, as those events are the most proximal to the activation of IGF1R. The next most sensitive parameters were *k*_*2*_ (IGF2-IGFBP association) and *k*_*8*_ (internalization of unbound IGF1R). Interestingly, the network was insensitive to all parameters regulating the interaction of IGF2 and IGF2R, as well as IGF2R trafficking. To determine if changes in these parameters impacted sensitivity to the level of IGF2R, a multivariate analysis was performed with a 10-fold increase in IGF2R in combination with each rate. To prevent offsetting effects, parameters were altered such that both perturbations resulted in a decrease in the level of pIGF1R (Fig. 6b). The ranking of the 10 most sensitive parameters did not change from the initial analysis and the impact on pIGF1R from a simultaneous change in parameters did not differ substantially from the sum of the effects of changing each rate and IGF2R individually. Combined, these analyses suggest there is no synergy between the rate parameters and sensitivity to IGF2R levels.

## Conclusions

The model presented here constitutes an important expansion of our foundational IGF model, which indicated that IGFBPs played an essential role in regulating IGF1-IGF1R interactions [27]. This expanded model incorporated the IGF2 axis of the IGF network (i.e.*,* IGF2 and IGF2R) and was used to assess the hypothesized roles of IGF2R and IGFBPs as tumor suppressors. Our model suggested, and experimental results confirmed, that IGF2R played only a minor role in counteracting IGF2 treatment. To significantly impact network activation, model analysis further suggested that IGF2R would need to be expressed at levels that are substantially higher than IGF1R and which do not appear to be physiologically relevant in several tumor types. In contrast to our analysis, a prior model of this system in cartilage suggested the IGF2R might play a role in regulating IGF2 interactions with IGF1R [28]; however, these findings were not confirmed experimentally and were not seen in our model utilizing data from cancer cells.

The inability of IGF2R to impact IGF1R signaling dynamics experimentally and under a variety of quantitative scenarios raises the question of why the *Igf2r−/−* mouse demonstrated a clear phenotype [15, 16] and what role IGF2R actually plays in the cellular network. It is of course possible that under conditions that were not included in these simulations, IGF2R can impact the IGF1R network. For example, despite IGFBP1 being the dominant IGFBP in fetal development [45, 46], the levels of IGF1BP in the fetal liver are only 4 % of the levels found in the maternal decidua [47]. Given the low levels of IGFBPs in fetal circulation, IGF2R could play a larger role in regulating circulating levels of IGF2 than is seen in the adult, explaining the effects seen in perinatal growth in the *Igf2r−/−* mouse. Additionally, the much longer timescale of fetal development may amplify the fairly modest effects seen in our simulations, resulting in a more important role for IGF2R. Finally, it is important to note that IGF2R has been shown to play a role in the cellular network beyond the IGF axis, as mannose-6-phosphate tagged proteins can also bind to IGF2R and be trafficked to the lysosome [48].

Importantly, the combined effect of the multiple genomic changes that lead to tumorigenesis could fundamentally change the parameter space and resulting behavior of the IGF network. For example, PTP1B, a phosphatase for IGF1R [49], is expressed at significantly lower levels in ovarian cancer cells compared to normal ovarian surface epithelial cells [50]. While ovarian surface epithelia are no longer considered to be the cell of origin for ovarian cancer [51], changes in PTP1B levels in combination with changes in the levels of other proteins could impact the kinetics of IGF1R activation. Likewise, a recent study demonstrated that clathrin-mediated, caveolae-mediated, and clathrin-independent endocytosis were all lower in non-small cell lung cancer cells compared to their normal bronchial epithelial counterparts [52]. Further, the rates for these processes also varied across a panel of non-small cell lung cancer cell lines. This report, combined with our finding that the IGF network was moderately sensitive to internalization rates suggests that analysis of cellular trafficking could prove useful to develop new therapeutic approaches.

While the model suggested a limited role for IGF2R, our analysis demonstrated that the high expression level of IGFBPs makes them a key control mechanism in response to IGF2 treatment, consistent with our prior findings that IGFBPs regulated IGF1-mediated network activation [27]. Additionally, due to their high expression level, IGFBPs were able to interfere with IGF2 treatment enough to slow cell proliferation. Our results demonstrate the potential for models of the IGF network to examine how system components interact to regulate behavior, which may help to identify optimal ways to target the IGF network for cancer therapy. In order to obtain a more complete understanding of the IGF system in carcinogenesis, future model refinement should focus on the function of individual IGFBPs, which can elicit different responses [17], and regulation of IGFBP-ligand interactions by cell-secreted proteases [53].

## Methods

### Cell culture and reagents

OVCAR5 cells were originally isolated from a serous ovarian carcinoma and were obtained from Dr. R. Bast (MD Anderson Cancer Center, Houston, TX). As an established member of the NCI-60 panel of cell lines, no additional ethics approval was needed. Cells were maintained at 37 °C in a humidified 5 % CO_2_ atmosphere in a complete culture medium composed of 1:1 (v/v) ratio of MCDB 105 and Medium 199 (Corning, Manassas, VA) supplemented with 10 % fetal bovine serum (Life Technologies, Carlsbad, CA) and 1 % penicillin/streptomycin. OVCAR5 cells were routinely tested and confirmed to be mycoplasma negative using the MycoAlert® Mycoplasma Detection Kit (Lonza, Rockland, ME). All reagents were from Sigma-Aldrich (St. Louis, MO) or Thermo Fisher Scientific (Waltham, MA) unless otherwise noted.

### Measurement of phosphorylated IGF1R (pIGF1R)

OVCAR5 were plated in 10 cm plates at 5700 cells/cm^2^, allowed to grow for 2 days, and then serum-starved for 24 h prior to treatment with recombinant human IGF1 or IGF2 (Peprotech, Rocky Hill, NJ). Cells were lysed at times ranging from 0 to 30 min after treatment with Bio-Plex lysis buffer (Bio-Rad Laboratories Inc., Hercules, CA). Total protein was measured by BCA assay and the level of pIGF1R was determined by the Bio-Plex pIGF1R (Tyr1131) assay on a Bio-Rad Bio-Plex 100 Suspension Array system (Bio-Rad Laboratories Inc.).

### siRNA knockdown of IGF1R and IGF2R

To knockdown IGF1R or IGF2R expression in OVCAR5, cells were plated in 35 mm plates at 5300 cells/cm^2^, allowed to attach for 6 h, and transfected for 24 h with 12.5 nM ON-TARGETplus Human IGF1R or IGF2R SMARTpool siRNA using DharmaFECT 1 siRNA Transfection Reagent (Dharmacon, Lafayette, CO). Transfected cells were changed to fresh complete media for 24 h, serum-starved for 24 h, and then treated with IGF ligands. Non-targeting siRNA was used as a negative control.

### Overexpression of IGF2R

pcDNA3.1(+)MPR-270, which expresses IGF2R under the CMV promoter, was kindly provided by Dr. William S. Sly (Saint Louis University, St. Louis, MO) [54]. OVCAR5 were plated in 35 mm plates at 5300 cells/cm^2^, allowed to grow for 24 h, and transfected with 2.5 μg DNA per plate using Lipofectamine® 3000 (Life Technologies) according to the manufacturer’s instructions. Transfection efficiency was determined to be 64 % by flow cytometry analysis of transfection with pEGFP. After 6 h of transfection, the media was changed to fresh complete media for 24 h, and then the cells were serum-starved for 24 h prior to IGF treatment. Transfection with pcDNA3.1(+) was used as a negative control.

### Quantification of cell proliferation

OVCAR5 were seeded in 12-well plates at 5300 cells/cm^2^, allowed to grow for 2 days, and then serum-starved for 24 h (resulting in a final density of 126,000 cells/well). IGF2 was then either spiked directly into conditioned media (which contains cell-secreted IGFBPs, [27]) or serum-free media was aspirated, cells were rinsed once with PBS, and the IGF2 treatment was added with fresh serum-free media. For experiments with IGFBP3, 15 nM of recombinant human IGFBP3 (Peprotech) was added 30 min prior to treatment with IGF2. All experiments were done with 1 mL of media per well. Cell proliferation was quantified after 24 h of IGF2 treatment using the Click-iT® EdU Alexa Fluor® 488 flow cytometry assay (Life Technologies) according to manufacturer’s instructions. Cells were incubated with EdU for 6 h prior to sample collection and analyzed on an Accuri C6 flow cytometer (BD, Franklin Lakes, NJ). To investigate the effects of IGF2R knockdown and overexpression on IGF2-induced proliferation, transfected cells were plated as described and treated with either vehicle or 1 nM IGF2.

### Mass-action kinetic model of IGF network

A mass-action kinetic model was developed to analyze the surface binding interactions between IGF ligands with IGFBPs, IGF1R, and IGF2R and the subsequent intracellular trafficking events (Fig. 1a). There were a total of 31 model parameters, of which 14 were determined experimentally or from published K_D_ values for IGF ligands with IGFBPs and IGF receptors (Additional files 1 and 2). The remaining 17 adjustable fitting parameters were determined by a least squares fit of the numerical solution of Eqs. 1a-x (Additional file 1) to experimental measurements of pIGF1R, using the lsqcurvefit fitting routine and ode45 solver in MATLAB v7.14 (MathWorks; Natick, MA). The lsqcurvefit routine minimizes the sum of the squares of the residual error between the model calculations and the experimental data, and the ode45 solver uses a variable step Runge-Kutta method. To ensure that the model found the global minimum, model fits were performed using 1000 randomly chosen initial guesses for the fitted parameters. For each of these combinations, the fitting routine returned to the same minimum within the fitting routine tolerance, suggesting this corresponds to a global minimum. Model analysis focused on the level of pIGF1R as IGF2-induced proliferation of OVCAR5 is dependent on IGF1R kinase activity (Additional file 6, [27]).

### Analysis of data from The Cancer Genome Atlas (TCGA)

RNA sequencing (RNA-Seq) data for ovarian serous cystadenocarcinoma, breast invasive carcinoma, and colon adenocarcinoma were obtained from TCGA (http://cancergenome.nih.gov/). To compare the individual and combined effectiveness of IGFBPs and IGF2R at inhibiting pIGF1R, model calculations were performed over a range of IGF2R:IGF1R and IGFBP:IGF1R ratios determined from the ovarian cancer data (one to the mean + one SD). This range encompasses greater than 80 % of the samples in the TCGA database. RNA-Seq data for normal breast and colon tissue were also obtained from the TCGA database; however, normal ovarian tissue data was not available.

### Sensitivity analysis

Sensitivity analysis was performed by increasing each parameter value and determining the effect on the integrated level of pIGF1R relative to the baseline condition. To determine if changes in the parameter set would alter the ability of IGF2R to regulate network activity, the sensitivity for each parameter altered in combination with a 10-fold increase in IGF2R was determined.

### Statistical analysis

All data are represented as the mean ± standard deviation and all experiments were performed with *n* = 3. Statistical significance was evaluated using student’s *t*-test analysis, with *p* < 0.05. All statistical calculations were performed using the software package JMP 4.1 (SAS Institute, Cary, NC).

### Availability of data

All data is available in the additional files or through the TCGA data portal https://tcga-data.nci.nih.gov/tcga/tcgaHome2.jsp.

## Additional files

Additional file 1:
**Detailed methods including the full set of differential equations and assumptions used in the model, experimental methods for determination of several rates and initial conditions, experimental methods for validation of the IGF2R knockdown and overexpression, and tables of both experimentally-determined and model-fitted rates and initial conditions.** (DOCX 80 kb) Additional file 2:
**Experimental results for the determination of internalization rates for IGF1-IGF1R, IGF2-IGF1R, IGF2-IGF2R.** (PDF 162 kb) Additional file 3:
**Experimental confirmation of IGF2R knockdown and overexpression.** (PDF 181 kb) Additional file 4:
**Analysis of the TCGA data for** ***IGF2R.*** (PDF 184 kb) Additional file 5:
**Analysis of the TCGA data for** ***IGFBPs.*** (PDF 197 kb) Additional file 6:
**Experimental validation of the impact of IGF1R and IR inhibition on IGF2-induced cell proliferation, demonstrating that IGF2-induced proliferation was dependent on IGF1R kinase activity and not IR kinase activity.** (PDF 104 kb)

## Abbreviations

IGF1: insulin-like growth factor 1
IGF2: insulin-like growth factor 2
IGFBPs: insulin-like growth factor binding proteins
IGF1R: type 1 insulin-like growth factor receptor
IGF2R: type 2 insulin-like growth factor receptor
IR: insulin receptor
