# Supplementary material for: Quantitative analysis of insulin-like growth factor 2 receptor and insulin-like growth factor binding proteins to identify control mechanisms for insulin-like growth factor 1 receptor phosphorylation
Source: BMC Syst Biol. 2016 Feb 9;10:15. doi: 10.1186/s12918-016-0263-6 (PMC4746774; doi:10.1186/s12918-016-0263-6)
Supplement: Additional file 1: — Detailed methods including the full set of differential equations and assumptions used in the model, experimental methods for determination of several rates and initial conditions, experimental methods for validation of the IGF2R knockdown and overexpression, and tables of both experimentally-determined and model-fitted rates and initial conditions. (DOCX 80 kb) [file 12918_2016_263_MOESM1_ESM.docx]

**Additional File 1**

**Supplementary Methods**

**Intracellular trafficking**

To determine the internalization rate of IGF1-IGF1R, OVCAR5 were plated in 35 mm plates at 5,300 cells/cm^2^, allowed to grow for 2 days, and then serum-starved for 24 hours prior to analysis of trafficking. To isolate the internalization rate of IGF2-IGF1R from IGF2-IGF2R, siRNA knockdown of IGF2R and IGF1R, respectively, was performed as described in the Materials and Methods. Trafficking rates were determined using established methods ([11](#_ENREF_11)). Briefly, serum-free media was aspirated, cells were rinsed once with PBS, and then incubated with 10 nM ^125^I-IGF ligand (PerkinElmer, Waltham, MA) at 37°C for 1.5, 3, 4.5, 6, 7.5, or 10 minutes. Binding was terminated by placing cells on ice and aspirating off the labeled ligand. Cells were then washed with ice-cold WHIPS solution (1 mg/mL polyvinylpyrrolidone, 130 mM NaCl, 5 mM KCl, 0.5 mM MgCl_2_, 1 mM CaCl_2_, 20 mM HEPES, pH 7.4) to remove unbound ^125^I-IGF. To obtain surface-associated ^125^I-IGF, cells were incubated in an acid strip solution (50 mM glycine-HCl, 100 mM NaCl, 1 mg/mL polyvinylpyrrolidone, 2 M urea, pH 3.0) for 8 minutes on ice. The acid strip solution was collected and the level of ^125^I-IGF was quantified by a gamma counter (Perkin-Elmer, Cobra II Auto-Gamma). To obtain internalized ^125^I-IGF, cells were solubilized with ice-cold 1 N NaOH for 8 minutes on ice, and this fraction was quantified by a gamma counter. The internalization rate for each ligand-receptor complex was determined as the slope of a plot of internalized ligand versus the integral of surface-associated ligand (Additional File 2) ([11](#_ENREF_11)).

To determine the endosomal exit rate and recycling fraction, OVCAR5 (transfected with siRNA against IGF1R or IGF2R for IGF2 trafficking experiments) were plated in 35 mm plates at 5,300 cells/cm^2^, allowed to grow for 2 days, and then serum-starved for 24 hours. Serum-free media was aspirated, cells were rinsed once with PBS, and then incubated with various concentrations of ^125^I-IGF ligand (0.1 to 10 nM) for 2 hours at 37°C to allow endosomal sorting to reach steady-state. After the incubation, OVCAR5 were washed with ice-cold WHIPS solution and incubated for 2 minutes with the acid strip solution without urea to remove surface-associated ligand. Cells were then washed with ice-cold WHIPS and returned to 37°C for 10 minutes in a saturating concentration (200 nM) of unlabeled IGF solution to inhibit the rebinding and reinternalization of recycled ^125^I-IGF. The medium was collected and centrifuged at 14,000 g for 20 minutes to separate degraded and intact (recycled) ligand in the medium using 3000 MWCO Amicon Ultra filter units (EMD Millipore, Billerica, MA). The flow through (degraded ligand) was separated from the filter which contained intact (recycled) ligand, and the radioactivity of the flow through and the filter were measured using a gamma counter. OVCAR5 were washed with WHIPS solution, incubated for 8 minutes with the acid strip solution containing urea to remove surface-associated ligand, and then solubilized with 1 N NaOH to collect internalized ligand. The endosomal exit rate was calculated using the internalized to surface-associated ligand distribution ([12](#_ENREF_12)). The recycling fraction was defined as the radioactivity of intact ligand in the medium divided by the radioactivity of the degraded and intact ligand combined ([12](#_ENREF_12)).

**Validation of IGF2R knockdown and overexpression**

OVCAR5 transfected with siRNA against IGF2R or pcDNA3.1(+) MPR-270 to overexpress IGF2R were lysed with whole cell lysis buffer composed of 6.3% glycerol, 2% SDS, 50 mM Tris-HCl (pH 6.8), 10 μg/mL aprotinin, 10 μg/mL leupeptin, 1 μg/mL pepstatin, 1 mM PMSF, 50 U/ml Benzonase nuclease, with freshly added Phosphatase Inhibitor II (5X total concentration; Boston BioProducts, Ashland, MA) and Phosphatase Inhibitor I (1X total concentration). Total protein was measured by BCA assay. Equal amounts of protein were separated by SDS–PAGE and blotted onto nitrocellulose membranes. Membranes were incubated overnight at 4°C in primary antibody and for 1 hour at room temperature in secondary antibody. Membranes were washed and fluorescence signals were detected and quantified using the Odyssey Infrared Imaging System (LI-COR Biotechnology, Lincoln, NE). Anti-IGF2R (1:50,000; ab124767) was purchased from Abcam (Cambridge, England) and anti-GAPDH (1:10,000; #2118) was purchased from Cell Signaling Technology (Devers, MA). Secondary antibodies were purchased from LI-COR Biotechnology and used at 1:15,000 dilution.

**Mass-action model of the IGF network**

A mass-action kinetics model was developed to analyze the surface binding interactions between IGF ligands with IGFBPs and IGF receptors and the subsequent intracellular trafficking events (Figure 1A). This model is described by the following system of ordinary differential equations:

Eqn. 1a $\frac{dC_{1}}{dt}=-k_{1}C_{1}C_{BP}+k_{-1}C_{1:BP}-k_{3}C_{1}C_{1R}+k_{-3}C_{1:1R}$

Eqn. 1b $\frac{dC_{2}}{dt}=-k_{2}{C_{2}C}_{BP}+k_{-2}C_{2:BP}-k_{4}C_{2}C_{2R}+k_{-4}C_{2:2R}-k_{5}C_{2}C_{1R}+k_{-5}C_{2:1R}$

Eqn. 1c $\frac{dC_{BP}}{dt}=-k_{1}C_{1}C_{BP}+k_{-1}C_{1:BP}-k_{2}C_{2}C_{BP}+k_{-2}C_{2:BP}$

Eqn. 1d $\frac{dC_{1R}}{dt}={-k}_{3}C_{1}C_{1R}+k_{-3}C_{1:1R}{-k}_{5}C_{2}C_{1R}+k_{-5}C_{2:1R}-k_{8}C_{1R}+k_{10}C_{1Rint}$

Eqn. 1e $\frac{dC_{2R}}{dt}={-k}_{4}C_{2}C_{2R}+k_{-4}C_{2:2R}-k_{11}C_{2R}+k_{13}C_{2Rint}$

Eqn. 1f $\frac{dC_{1:BP}}{dt}=k_{1}C_{1}C_{BP}-k_{-1}C_{1:BP}$

Eqn. 1g $\frac{dC_{2:BP}}{dt}=k_{2}C_{2}C_{BP}-k_{-2}C_{2:BP}$

Eqn. 1h $\frac{dC_{1:1R}}{dt}=k_{3}C_{1}C_{1R}-k_{-3}C_{1:1R}+k_{-6}C_{1:1R*}-k_{6}C_{1:1R}+{f_{1}k}_{15}C_{1:1Rint}$

Eqn. 1i $\frac{dC_{2:1R}}{dt}=k_{5}C_{2}C_{1R}-k_{-5}C_{2:1R}+k_{-7}C_{2:1R*}-k_{7}C_{2:1R}+{f_{2}k}_{17}C_{2:1Rint}$

Eqn. 1j $\frac{dC_{2:2R}}{dt}=k_{4}C_{2}C_{2R}-k_{-4}C_{2:2R}-k_{18}C_{2:2R}+{f_{3}k}_{19}C_{2:2Rint}$

Eqn. 1k $\frac{dC_{1:1R*}}{dt}={-k}_{-6}C_{1:1R*}+k_{6}C_{1:1R}-k_{14}C_{1:1R*}$

Eqn. 1l $\frac{dC_{2:1R*}}{dt}={-k}_{-7}C_{2:1R*}+k_{7}C_{2:1R}-k_{16}C_{2:1R*}$

Eqn. 1m $\frac{dC_{1:1Rint}}{dt}=k_{20}C_{1:1R*}-{\left( 1-f_{1} \right)k}_{15}C_{1:1Rint}-{f_{1}k}_{15}C_{1:1Rint}$

Eqn. 1n $\frac{dC_{2:1Rint}}{dt}=k_{21}C_{2:1R*}-{\left( 1-f_{2} \right)k}_{17}C_{2:1Rint}-{f_{2}k}_{17}C_{2:1Rint}$

Eqn. 1o $\frac{dC_{1:1R*int}}{dt}={k_{14}C}_{1:1R*}-k_{20}C_{1:1R*int}$

Eqn. 1p $\frac{dC_{2:1R*int}}{dt}={k_{16}C}_{2:1R*}{-k}_{21}C_{2:1R*int}$

Eqn. 1q $\frac{dC_{2:2Rint}}{dt}={k_{18}C}_{2:2R}{-\left( 1-f_{3} \right)k}_{19}C_{2:2Rint}{-f_{3}k}_{19}C_{2:2Rint}$

Eqn. 1r $\frac{dC_{1:1Rdeg}}{dt}={\left( 1-f_{1} \right)k}_{15}C_{1:1Rint}$

Eqn. 1s $\frac{dC_{2:1Rdeg}}{dt}={\left( 1-f_{2} \right)k}_{17}C_{2:1Rint}$

Eqn. 1t $\frac{dC_{2:2Rdeg}}{dt}={\left( 1-f_{3} \right)k}_{19}C_{2:2Rint}$

Eqn. 1u $\frac{dC_{1Rint}}{dt}={k_{8}C}_{1R}{-k}_{9}C_{1Rint}{-k}_{10}C_{1Rint}$

Eqn. 1v $\frac{dC_{2Rint}}{dt}={k_{11}C}_{2R}{-k}_{12}C_{2Rint}{-k}_{13}C_{2Rint}$

Eqn. 1w $\frac{dC_{1Rdeg}}{dt}=k_{9}C_{2Rint}$

Eqn. 1x $\frac{dC_{2Rdeg}}{dt}=k_{12}C_{2Rdeg}$

where *C_i_* is the concentration of component *i* and the subscripts *1*, *2*, *1R*, *2R*, and *BP* refer to IGF1, IGF2, IGF1R, IGF2R, and IGFBP, respectively. A list of model species and their corresponding symbols is presented in Methods Table 1. This model expanded upon our previous foundational IGF model ([13](#_ENREF_13)). Several assumptions were utilized in the development of this expanded model. First, the model assumes reversible binding interactions between IGF ligands and IGFBPs, and between IGF ligands and IGF receptors. Phosphorylation of IGF1R was assumed to be reversible, while intracellular trafficking events were irreversible. Second, the binding affinity of all six structurally related IGFBPs for IGF ligands have been reported to be within the same order of magnitude ([1](#_ENREF_1)); therefore, for model simplification IGFBP1-6 were consolidated into one term. While IGFBPs under certain conditions can potentiate IGF action ([14](#_ENREF_14)), we assumed that the sole action of IGFBPs *in vitro* was to sequester IGF ligands from binding to IGF receptors and that IGFBPs do not degrade. Third, the model did not include IR or IGF1R:IR as IGF-induced proliferation was determined to be independent of IR kinase activity (Additional File 6, ([13](#_ENREF_13))) and previous analysis in our lab demonstrated that less than 10% of IGF1R was incorporated into heterodimers in OVCAR5 ([13](#_ENREF_13)). Fourth, we assumed IGF1R can traffic without IGF ligand and that once IGF ligand is bound to IGF1R, the receptor needs to be phosphorylated in order to undergo internalization. IGF2R can traffic without IGF ligand and once ligand bound, IGF2-IGF2R can also traffic. Fifth, due to the short experimental time scale, synthesis of IGF receptor and IGFBP were assumed to be negligible ([13](#_ENREF_13),[15](#_ENREF_15),[16](#_ENREF_16)). Lastly, internalized and surface phosphorylated IGF1-IGF1R and IGF2-IGF1R both contributed to the measured level of pIGF1R, as receptor tyrosine kinases have been observed to remain phosphorylated after internalization ([17-20](#_ENREF_17)) and our experimental method was not cell compartment specific. IGF1-IGF1R and IGF2-IGF1R were assumed to only recycle once dephosphorylated.

Initial conditions were set to zero for complexes and IGF ligand concentration was determined from the treatment conditions. The initial concentration of IGF1R per cell and IGFBPs per cell for OVCAR5 were previously determined ([13](#_ENREF_13)). The initial concentration of IGF2R per cell was measured using a total-IGF2R ELISA assay (R&D Systems, Minneapolis, MN). To convert pIGF1R measurements into units appropriate for the model, we assumed that the maximal observed pIGF1R level corresponded to complete saturation ([21](#_ENREF_21)). Initial conditions and rate parameters are presented in Methods Tables 2 and 3.

**Methods Table 1.** **Model species and symbols.**

| Model Species | Symbol |
| --- | --- |
| IGF1 | 1 |
| IGF2 | 2 |
| IGFBP | BP |
| IGF1R | 1R |
| IGF2R | 2R |
| IGF1:IGFBP | 1:BP |
| IGF2:IGFBP | 2:BP |
| IGF1:IGF1R | 1:1R |
| IGF2:IGF1R | 2:1R |
| IGF2:IGF2R | 2:2R |
| IGF1:pIGF1R | 1:1R^*^ |
| IGF2:pIGF1R | 2:1R^*^ |
| IGF1:pIGF1R_int_ | 1:1R^*^_int_ |
| IGF2:pIGF1R_int_ | 2:1R^*^_int_ |
| IGF2:IGF2R_int_ | 2:2R_int_ |
| IGF1:IGF1R_int_ | 1:1R_int_ |
| IGF2:IGF1R_int_ | 2:1R_int_ |
| IGF1:IGF1R_deg_ | 1:1R_deg_ |
| IGF2:IGF1R_deg_ | 2:1R_deg_ |
| IGF2:IGF2R_deg_ | 2:2R_deg_ |
| IGF1R_int_ | 1R_int_ |
| IGF2R_int_ | 2R_int_ |
| IGF1R_deg_ | 1R_deg_ |
| IGF2R_deg_ | 2R_deg_ |

**Methods Table 2. Experimentally-determined initial conditions for OVCAR5.**

| Initial Condition | Value |
| --- | --- |
| IGFBPs | 1.21×10^-8^ nmol cell^-1^ |
| IGF1R | 2.23×10^-11^ nmol cell^-1^ |
| IGF2R | 1.53×10^-11^ nmol cell^-1^ |

| Methods Table 3. Values for rate coefficients. |  |  |
| --- | --- | --- |
| Rate Coefficient | **Symbol** | **Value** |
| Association rate coefficient of IGF1:IGFBP complex^a^ | *k_1_* | 1.162x10^8^ nmol^-1^ cell min^-1^ |
| Dissociation rate coefficient of IGF1 and IGFBP^b^ | *k_-1_* | 0.042 min^-1^ |
| Association rate coefficient of IGF2:IGFBP complex^a^ | *k_2_* | 4.734x10^7^ nmol^-1^ cell min^-1^ |
| Dissociation rate coefficient of IGF2 and IGFBP^b^ | *k_-2_* | 0.017 min^-1^ |
| Association rate coefficient of IGF1:IGF1R complex^a^ | *k_3_* | 8.797x10^8^ nmol^-1^ cell min^-1^ |
| Dissociation rate coefficient of IGF1 and IGF1R^b^ | *k_-3_* | 3.167 min^-1^ |
| Association rate coefficient of IGF2:IGF2R complex^a^ | *k_4_* | 9.494x10^8^ nmol^-1^ cell min^-1^ |
| Dissociation rate coefficient of IGF2 and IGF2R^b^ | *k_-4_* | 3.418 min^-1^ |
| Association rate coefficient of IGF2:IGF1R complex^a^ | *k_5_* | 3.561x10^7^ nmol^-1^ cell min^-1^ |
| Dissociation rate coefficient of IGF2 and IGF1R^b^ | *k_-5_* | 0.128 min^-1^ |
| Phosphorylation rate coefficient of IGF1:IGF1R^b^ | *k_6_* | 0.704 min^-1^ |
| Dephosphorylation rate coefficient of IGF1:pIGF1R^b^ | *k_-6_* | 0.138 min^-1^ |
| Phosphorylation rate coefficient of IGF2:IGF1R^b^ | *k_7_* | 1.967 min^-1^ |
| Dephosphorylation rate coefficient of IGF2:pIGF1R^b^ | *k_-7_* | 3.317 min^-1^ |
| Internalization rate coefficient of unbound IGF1R^b^ | *k_8_* | 0.359 min^-1^ |
| Degradation rate coefficient of unbound IGF1R_int_^b^ | *k_9_* | 1.745 min^-1^ |
| Recycling rate coefficient of unbound IGF1R_int_^b^ | *k_10_* | 0.003 min^-1^ |
| Internalization rate coefficient of unbound IGF2R^b^ | *k_11_* | 0.936 min^-1^ |
| Degradation rate coefficient of unbound IGF2R_int_^b^ | *k_12_* | 0.171 min^-1^ |
| Recycling rate coefficient of unbound IGF2R_int_^b^ | *k_13_* | 10.478 min^-1^ |
| Internalization rate coefficient of IGF1:pIGF1R^c^ | *k_14_* | 0.023 min^-1^ |
| Endosomal exit rate coefficient of IGF1:IGF1R_int_^c^ | *k_15_* | 0.031 min^-1^ |
| Internalization rate coefficient of IGF2:pIGF1R^c^ | *k_16_* | 0.022 min^-1^ |
| Endosomal exit rate coefficient of IGF2:IGF1R_int_^c^ | *k_17_* | 0.045 min^-1^ |
| Internalization rate coefficient of IGF2:IGF2R^c^ | *k_18_* | 0.036 min^-1^ |
| Endosomal exit rate coefficient of IGF2:IGF2R_int_^c^ | *k_19_* | 0.055 min^-1^ |
| Dephosphorylation rate coefficient of internalized IGF1:pIGF1R^b^ | *k_20_* | 3.260 min^-1^ |
| Dephosphorylation rate coefficient of internalized IGF2:pIGF1R^b^ | *k_21_* | 1.962 min^-1^ |
| Recycling fraction of IGF1:pIGF1R_int_^c^ | *f_1_* | 0.92 |
| Recycling fraction of IGF2:pIGF1R_int_^c^ | *f_2_* | 0.97 |
| Recycling fraction of IGF2:IGF2R_int_^c^ | *f_3_* | 0.93 |

^a^Evaluated from dissociation constants (*K_d_*) obtained from literature and the fitted value of the corresponding dissociation rate coefficient ([1-10](#_ENREF_1)).

^b^Determined from model fitting.

^c^Experimentally determined for OVCAR5 cells.

**References**

1. Hwa V, Oh Y, Rosenfeld RG. The insulin-like growth factor-binding protein (IGFBP) superfamily. Endocrine reviews 1999;20(6):761-87.

2. Beattie J, Allan GJ, Lochrie JD, Flint DJ. Insulin-like growth factor-binding protein-5 (IGFBP-5): a critical member of the IGF axis. The Biochemical journal 2006;395(1):1-19.

3. Shin SU, Friden P, Moran M, Morrison SL. Functional properties of antibody insulin-like growth factor fusion proteins. The Journal of biological chemistry 1994;269(7):4979-85.

4. Pommier GJ, Remacle-Bonnet MM, Tripier SG, Garrouste FL. Differential secretory polarity of IGFBP-6 vs. IGFBP-2 and IGFBP-4 in human intestinal epithelial cells: is it a way of modulating IGF-II bioavailability towards the IGF-responsive basolateral surface? Progress in growth factor research 1995;6(2-4):197-206.

5. Accili D, Nakae J, Kim JJ, Park BC, Rother KI. Targeted gene mutations define the roles of insulin and IGF-I receptors in mouse embryonic development. Journal of pediatric endocrinology & metabolism : JPEM 1999;12(4):475-85.

6. Jurgeit A, Berlato C, Obrist P, Ploner C, Massoner P, Schmolzer J, et al. Insulin-like growth factor-binding protein-5 enters vesicular structures but not the nucleus. Traffic 2007;8(12):1815-28.

7. Robinson SA, Rosenzweig SA. Synthesis and characterization of biotinylated forms of insulin-like growth factor-1: topographical evaluation of the IGF-1/IGFBP-2 AND IGFBP-3 interface. Biochemistry 2004;43(36):11533-45.

8. Danielsen A, Larsen E, Gammeltoft S. Chromaffin cells express two types of insulin-like growth factor receptors. Brain research 1990;518(1-2):95-100.

9. Germain-Lee EL, Janicot M, Lammers R, Ullrich A, Casella SJ. Expression of a type I insulin-like growth factor receptor with low affinity for insulin-like growth factor II. The Biochemical journal 1992;281 ( Pt 2):413-7.

10. Lammers R, Gray A, Schlessinger J, Ullrich A. Differential signalling potential of insulin- and IGF-1-receptor cytoplasmic domains. EMBO J 1989;8(5):1369-75.

11. Wiley HS, Herbst JJ, Walsh BJ, Lauffenburger DA, Rosenfeld MG, Gill GN. The role of tyrosine kinase activity in endocytosis, compartmentation, and down-regulation of the epidermal growth factor receptor. The Journal of biological chemistry 1991;266(17):11083-94.

12. French AR, Tadaki DK, Niyogi SK, Lauffenburger DA. Intracellular trafficking of epidermal growth factor family ligands is directly influenced by the pH sensitivity of the receptor/ligand interaction. The Journal of biological chemistry 1995;270(9):4334-40.

13. Tian D, Kreeger PK. Analysis of the quantitative balance between insulin-like growth factor (IGF)-1 ligand, receptor, and binding protein levels to predict cell sensitivity and therapeutic efficacy. BMC systems biology 2014;8(1):98.

14. Miyake H, Nelson C, Rennie PS, Gleave ME. Overexpression of insulin-like growth factor binding protein-5 helps accelerate progression to androgen-independence in the human prostate LNCaP tumor model through activation of phosphatidylinositol 3'-kinase pathway. Endocrinology 2000;141(6):2257-65.

15. Creek KE, Sly WS. Biosynthesis and turnover of the phosphomannosyl receptor in human fibroblasts. The Biochemical journal 1983;214(2):353-60.

16. Sahagian GG, Neufeld EF. Biosynthesis and turnover of the mannose 6-phosphate receptor in cultured Chinese hamster ovary cells. The Journal of biological chemistry 1983;258(11):7121-8.

17. Wada I, Lai WH, Posner BI, Bergeron JJ. Association of the tyrosine phosphorylated epidermal growth factor receptor with a 55-kD tyrosine phosphorylated protein at the cell surface and in endosomes. The Journal of cell biology 1992;116(2):321-30.

18. Sorkin A, Eriksson A, Heldin CH, Westermark B, Claesson-Welsh L. Pool of ligand-bound platelet-derived growth factor beta-receptors remain activated and tyrosine phosphorylated after internalization. Journal of cellular physiology 1993;156(2):373-82.

19. Wiley HS. Trafficking of the ErbB receptors and its influence on signaling. Experimental cell research 2003;284(1):78-88.

20. Chow JC, Condorelli G, Smith RJ. Insulin-like growth factor-I receptor internalization regulates signaling via the Shc/mitogen-activated protein kinase pathway, but not the insulin receptor substrate-1 pathway. The Journal of biological chemistry 1998;273(8):4672-80.

21. Chen WW, Schoeberl B, Jasper PJ, Niepel M, Nielsen UB, Lauffenburger DA, et al. Input-output behavior of ErbB signaling pathways as revealed by a mass action model trained against dynamic data. Molecular systems biology 2009;5:239.
