# Supplementary material for: Quantitative analysis of insulin-like growth factor 2 receptor and insulin-like growth factor binding proteins to identify control mechanisms for insulin-like growth factor 1 receptor phosphorylation
Source: BMC Syst Biol. 2016 Feb 9;10:15. doi: 10.1186/s12918-016-0263-6 (PMC4746774; doi:10.1186/s12918-016-0263-6)
Supplement: Additional file 2: — Experimental results for the determination of internalization rates for IGF1-IGF1R, IGF2-IGF1R, IGF2-IGF2R. (PDF 162 kb) [file 12918_2016_263_MOESM2_ESM.pdf]

Additional File 2

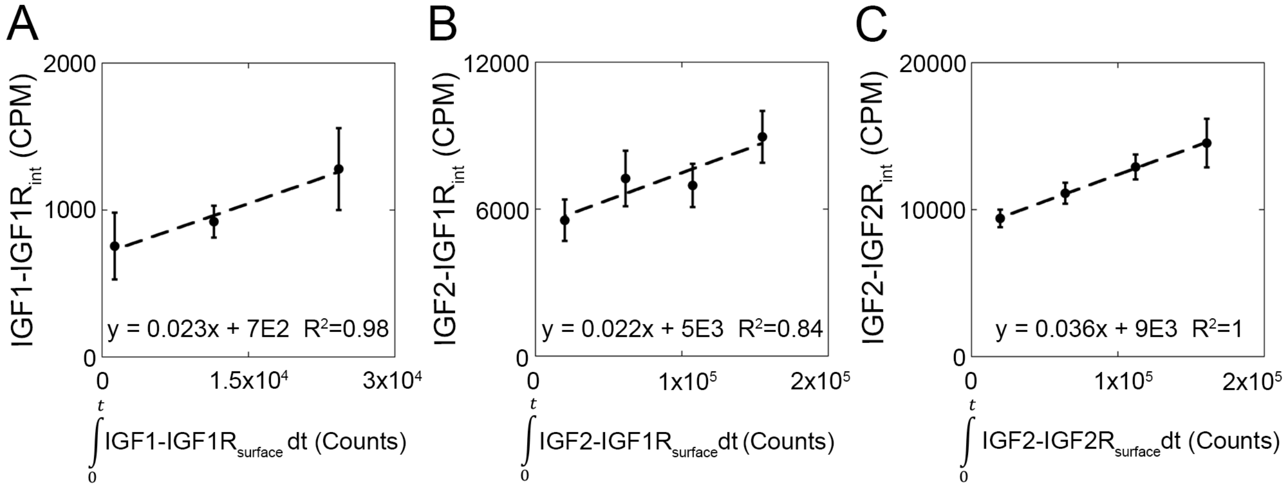

Internalization rates for IGF receptors in OVCAR5. The internalization rates for IGF1-IGF1R (***A***), IGF2-IGF1R (***B***), and IGF2-IGF2R (***C***) were determined from the slope of the internalized ligand versus the integrated surface-associated ligand.
