# Supplementary material for: Quantitative analysis of insulin-like growth factor 2 receptor and insulin-like growth factor binding proteins to identify control mechanisms for insulin-like growth factor 1 receptor phosphorylation
Source: BMC Syst Biol. 2016 Feb 9;10:15. doi: 10.1186/s12918-016-0263-6 (PMC4746774; doi:10.1186/s12918-016-0263-6)
Supplement: Additional file 3: — Experimental confirmation of IGF2R knockdown and overexpression. (PDF 181 kb) [file 12918_2016_263_MOESM3_ESM.pdf]

Additional File 3

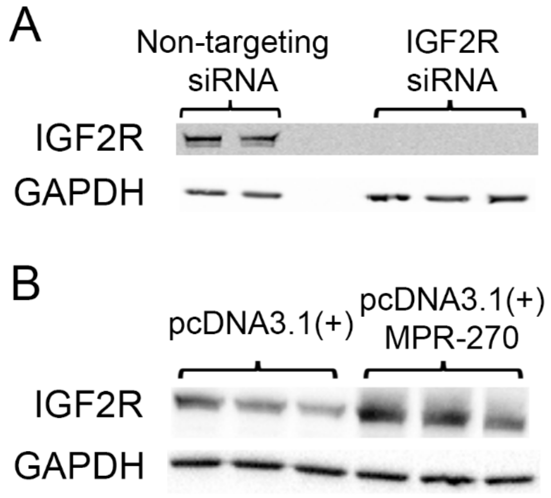

Western blot confirmation of IGF2R knockdown and overexpression in OVCAR5. (***A***) siRNA knockdown of IGF2R was greater than 90% in OVCAR5. (***B***) IGF2R was overexpressed by 1.4-fold in OVCAR5 transfected with pcDNA3.1(+)MPR-270. n=3 per condition.
