# Supplementary material for: Quantitative analysis of insulin-like growth factor 2 receptor and insulin-like growth factor binding proteins to identify control mechanisms for insulin-like growth factor 1 receptor phosphorylation
Source: BMC Syst Biol. 2016 Feb 9;10:15. doi: 10.1186/s12918-016-0263-6 (PMC4746774; doi:10.1186/s12918-016-0263-6)
Supplement: Additional file 5: — Analysis of the TCGA data for IGFBPs. (PDF 197 kb) [file 12918_2016_263_MOESM5_ESM.pdf]

Additional File 5

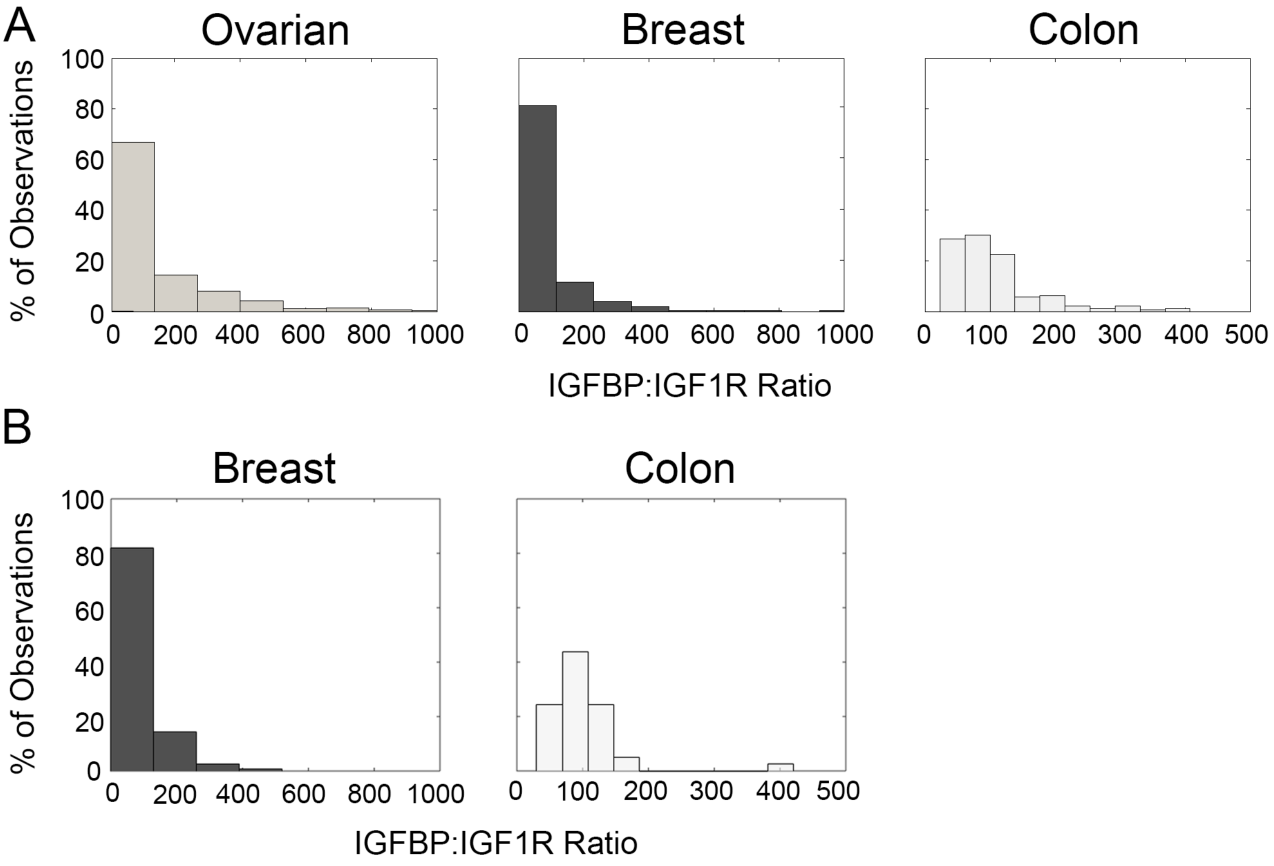

Analysis of the TCGA data for *IGFBP*. *(A)* Expression of *IGFBP* relative to *IGF1R* in ovarian serous cystadenocarcinoma, breast invasive carcinoma, and colon adenocarcinoma. ***(B)*** Expression of *IGFBP* relative to *IGF1R* in normal breast and colon tissue. There was no significant difference in IGFBP:IGF1R ratio between normal tissue and tumor for both colon and breast cancer. Note differences in x-axis scales.
