# Supplementary material for: Quantitative analysis of insulin-like growth factor 2 receptor and insulin-like growth factor binding proteins to identify control mechanisms for insulin-like growth factor 1 receptor phosphorylation
Source: BMC Syst Biol. 2016 Feb 9;10:15. doi: 10.1186/s12918-016-0263-6 (PMC4746774; doi:10.1186/s12918-016-0263-6)
Supplement: Additional file 6: — Experimental validation of the impact of IGF1R and IR inhibition on IGF2-induced cell proliferation, demonstrating that IGF2-induced proliferation was dependent on IGF1R kinase activity and not IR kinase activity. (PDF 104 kb) [file 12918_2016_263_MOESM6_ESM.pdf]

Additional File 6

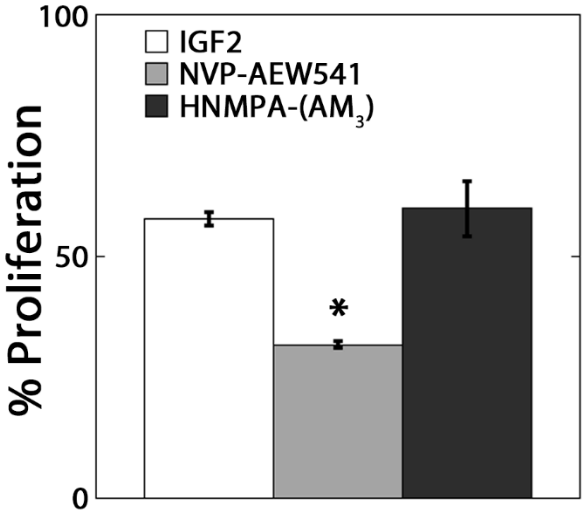

OVCAR5 proliferation in response to IGF2 was dependent on IGF1R kinase activity. Cells were pre-treated with an IGF1R tyrosine kinase inhibitor (NVP-AEW541, 1 μM) or an IR tyrosine kinase inhibitor (HNMPA-(AM_3), 5 μM) for 30 minutes before stimulation with a saturating dose of IGF2 (13 nM) for 24 hours. The results demonstrated that IGF1R kinase activity was essential for OVCAR5 proliferation in response to IGF2, while IR kinase activity was not. * indicates significantly different ($p<0.05$) from IGF2-treated, n=3 per treatment.
